# Dapaglifozin on Albuminuria in Chronic Kidney Disease Patients with FabrY Disease: The DEFY Study Design and Protocol

**DOI:** 10.3390/jcm12113689

**Published:** 2023-05-26

**Authors:** Yuri Battaglia, Francesca Bulighin, Luigi Zerbinati, Nicola Vitturi, Giacomo Marchi, Gianni Carraro

**Affiliations:** 1 Department of Medicine, University of Verona, 37129 Verona, Italy; 2 Nephrology and Dialysis Unit, Pederzoli Hospital, 37019 Peschiera del Garda, Italy; 3 Institute of Psychiatry, Department of Neuroscience and Rehabilitation, University of Ferrara, Via Fossato di Mortara 64a, 44100 Ferrara, Italy; 4 Department of Medicine-DIMED, Division of Metabolic Diseases, University Hospital, 35128 Padova, Italy; 5 Department of Medicine, Section of Internal Medicine, University of Verona, 37129 Verona, Italy; 6 MetabERN Referral Center, Azienda Ospedaliera Universitaria Integrata, 37129 Verona, Italy; 7 Nephrology, Dialysis and Transplantation Unit, Department of Medicine, University of Padova, 35128 Padova, Italy

**Keywords:** SGLT2 inhibitors, nephropathy, chronic kidney disease, migalastat, ACE inhibitors, angiotensin receptor blockers

## Abstract

Fabry disease (FD) is a rare genetic disorder caused by a deficiency in the α-galactosidase A enzyme, which results in the globotriaosylceramide accumulation in many organs, including the kidneys. Nephropathy is a major FD complication that can progress to end-stage renal disease if not treated early. Although enzyme replacement therapy and chaperone therapy are effective, other treatments such as ACE inhibitors and angiotensin receptor blockers can also provide nephroprotective effects when renal damage is also established. Recently, SGLT2 inhibitors have been approved as innovative drugs for treating chronic kidney disease. Thus, we plan a multicenter observational prospective cohort study to assess the effect of Dapagliflozin, a SGLT2 inhibitor, in FD patients with chronic kidney disease (CKD) stages 1–3. The objectives are to evaluate the effect of Dapagliflozin primarily on albuminuria and secondarily on kidney disease progression and clinical FD stability. Thirdly, any association between SGT2i and cardiac pathology, exercise capacity, kidney and inflammatory biomarkers, quality of life, and psychosocial factors will also be evaluated. The inclusion criteria are age ≥ 18; CKD stages 1–3; and albuminuria despite stable treatment with ERT/Migalastat and ACEi/ARB. The exclusion criteria are immunosuppressive therapy, type 1 diabetes, eGFR < 30 mL/min/1.73 m^2^, and recurrent UTIs. Baseline, 12-month, and 24-month visits will be scheduled to collect demographic, clinical, biochemical, and urinary data. Additionally, an exercise capacity and psychosocial assessment will be performed. The study could provide new insights into using SGLT2 inhibitors for treating kidney manifestations in Fabry disease.

## 1. Introduction

Fabry disease (FD) is a rare X-linked lysosomal storage disorder characterized by a deficiency in the activity of lysosomal α-galactosidase-A. This genetic mutation leads to the accumulation of globotriaosylceramide (Gb3) in various cell types, including endothelial, cardiac, renal, and neuronal cells, resulting in progressive damage to the renal, cardiac, and nervous system [1]. Kidney biopsies typically reveal Gb3 accumulation primarily in podocytes, mesangial cells, and tubular epithelial cells, with focal and global glomerulosclerosis usually starting in the second decade of life or even earlier in some cases [2,3]. It is worth noting that Gb3 accumulation alone is not solely responsible for the disease’s pathogenesis [4]. Inflammation and oxidative stress also contribute to organ damage, potentially explaining the suboptimal response of enzyme replacement therapy (ERT) in cases of late-disease diagnosis [5].

Clinically, three presentations of FD can be identified [1]. In hemizygous males with the classic form, the disease typically begins in childhood and presents with symptoms such as acroparesthesias, angiokeratomas, anhidrosis, cornea verticillate, and gastrointestinal disorders. Cardiac hypertrophy, arrhythmias, stroke, and chronic kidney disease usually appear later in life [6,7,8,9,10]. For heterozygous females, the clinical manifestations can vary widely, ranging from asymptomatic to mild or severe forms, and depend on the X-inactivation process. The FD later-onset form typically appears between the fourth and seventh decade of life and predominantly affects the heart or kidneys [11].

Nephropathy is a major FD complication, characterized by a reduced glomerular filtration rate and proteinuria. In the absence of a specific therapy, impaired renal function progressively worsens over time, leading to end-stage renal disease being reached around the fourth or fifth decade of life. Proteinuria is the primary predictor for renal progression in both men and women with FD [12]. Additionally, proteinuria is a risk factor for cardiovascular events in patients with chronic kidney disease (CKD) and, therefore, is an important therapeutic target.

The FD treatment involves specific therapy, such as enzyme replacement therapy using Agalsidase alpha/Agalsidase beta and chaperone therapy using Migalastat [13], which are aimed at managing organ damage [14,15]. However, there have been debates and conflicting results regarding the effect of these specific FD therapies on proteinuria in FD patients with CKD. Specifically, the use of Agalsidase alpha did not show any anti-proteinuric effect, while the reduction in proteinuria across clinical trials with Agalsidase beta varied significantly [16]. Furthermore, preliminary studies indicate conflicting results regarding the reduction in proteinuria with Migalastat [17].

In addition to the above therapies, other drugs such as ACE inhibitors (ACEi) and angiotensin receptor blockers (ARB) play a relevant nephroprotective role by reducing proteinuria and slowing the decline in the glomerular filtration rate (GFR). To achieve a nephroprotective effect, doses of ACEi/ARBs must be titrated to reduce proteinuria up to 0.5 g/24 h [18].

However, the use of ACEi or ARBs is limited due to the risk of hyperkalemia and acute kidney injury. Although a double blockade of ACEi and ARB demonstrated a greater antiproteinuric effect in patients with or without diabetes and with microalbuminuria or proteinuria, it is not recommended due to increased adverse events [19]. Furthermore, there is a lack of data available regarding the use of double blockade in Fabry nephropathy. 

SGLT2 inhibitors (SGLT2i), initially introduced as hypoglycaemic drugs, have become increasingly popular in recent years for the treatment of CKD and heart failure, regardless of glycaemic control. These drugs act by inhibiting the sodium–glucose cotransporters channels present in the S1 tract of the proximal tubule, which are responsible for the majority of filtered glucose reabsorption. Several large randomized controlled trials showed the benefits of SGLT2i in both cardiac and renal function, not only in patients with diabetes but also in patients without diabetes [20]. Indeed, SGLT2 inhibitors reduce the risk of cardiovascular death and hospitalization for heart failure similarly in patients with diabetes mellitus, hypertensive nephropathy, and glomerulonephritis. [21]. On the other hand, the results of two recent trials, such as EMPA-KIDNEY [22] and DELIVER [23], conducted in patients with or without diabetes mellitus have demonstrated that the use of SGLT2i reduced the risk of CKD progression (37%) and of acute kidney injury (AKI) onset (23%). These renal benefits were observed regardless of the underlying nephropathy or stage of glomerular filtrate rate [22]. An analysis of the DAPA-CKD trial [24] showed that dapagliflozin can significantly reduce albuminuria in CKD patients with and without diabetes mellitus. Consequently, SGLT2 inhibitors have been recently approved in CKD patients with proteinuria for the treatment of CKD progression [25].

SGLT2 inhibitors exert their nephroprotective effect through multiple mechanisms, including the re-establishment of glomerular tubular feedback, which protects against glomerular hyperfiltration damage. In depth, this involves the reduction in glucose and sodium reabsorption in the proximal tubule leading to increased sodium transport at the macula densa, increased adenosine release, afferent arteriole vasoconstriction, and a reduction in intraglomerular pressure [26]. These effects could be added to those of ACEi/ARBs, which act at the level of the efferent arteriole, causing vasodilation. 

In addition to these mechanisms, other factors can contribute to the nephroprotective effects of SGLT2 inhibitors, including the reduction in blood pressure values, an increase in hemoglobin and hematocrit values, a decrease in uric acid levels, and a reduction in oxidative stress and pro-inflammatory mediators [27,28]. 

These benefits suggest a potential role for SGLT2i in FD patients with CKD as these drugs could target the FD pathogenetic mechanisms, such as oxidative stress and inflammation, which are not completely addressed by ERT or chaperon therapy alone. However, further investigations and well-designed clinical trials are needed to thoroughly assess the effects of SGLT2i on Fabry nephropathy [29].

## 2. Aim of Study

With this background in mind, we have planned a multicenter observational prospective cohort study involving some Italian Nephrology and Internal Medicine Units. The study aims to evaluate the effect of an SGLT2 inhibitor, Dapaglifozin, in Fabry disease patients with CKD stages 1–3. Specifically, the primary objective will be to determine the effect of Dapaglifozin on albuminuria, with a target of achieving a 20% reduction in albuminuria. As secondary aim, we will assess the effect of Dapaglifozin on kidney disease progression, measured by a reduction in eGFR < 3 mL/min/1.73 m^2^ per year and on clinical FD stability, defined by a FAbry STabilization indEX (FASTEX) score < 20 [30]. Additionally, any association of SGT2i with the progression of cardiac pathology, exercise capacity, kidney and inflammatory biomarkers, quality of life, and psychosocial factors will be evaluated.

## 3. Methods

The study was approved by the Hospital Ethics Committee for Clinical Trials CESC (Ref No. 5499/AO/22). The primary and secondary end-points will include albuminuria, estimated GFR calculated using the CKD-EPI formula, septum ventricular diameter and diastolic function detected at echocardiography, left ventricular mass index/m^2^ and evolution of T1 mapping pattern at magnetic resonance imaging (MRI), FASTEX score, 5 Times Sit to Stand Test (5STS) [31], 6 Minute Walking Test (6MWT) [32], Cardiopulmonary Exercise Testing (CPET), quality of life and psychosocial questionnaires, and urinary tract infections. In addition, the dosage of exomes, IgG, IgG4, kidney injury molecule-1 (KIM-1), neutrophil-gelatinase-associated lipocalin (NGAL), liver-fatty-acid-binding protein (LFABP), isoprostane, tumor necrosis factor alpha (TNF-alpha), interleukin-1 (IL-1), and interleukin-6 (IL-6) will be tested. 

The study will enroll male and female subjects aged 18 years or older with CKD stages 1–3, a genetic and biochemical diagnosis of FD and albuminuria for at least 6 months despite treatment with a stable dose of ERT or Migalastat for at least 12 months and ACEi or ARB titrated to the maximum tolerated dosage for at least 6 months. The subjects with immunosuppressive therapy, type 1 diabetes mellitus, eGFR < 30 mL/min/1.73 m^2^, and recurrent urinary tract infections will be excluded. 

On the basis of previous evidence [24], we estimated that at least 28 patients would need to be recruited to answer the research question with a 95% level of confidence and a margin of error of 5%.

The study will last two years and will include baseline, 12-month, and 24-month visits. 

At the baseline visit, the demographic data (age, sex), anamnestic data (comorbidities, medications, underling kidney disease), and clinical parameters (blood pressure, heart rate, height, weight, Body Mass Index [BMI]) will be collected. Biochemical examinations (LysoGb3, hematocrit, hemoglobin, fasting glucose, creatinine, urea, cystatin C, sodium, potassium, calcium, phosphorus, uric acid, total proteins, albumin, vitamin D, parathormone, brain natriuretic peptide [BNP], troponin I, polymerase chain reaction [PCR], exomes, IgG, IgG4, KIM-1, NGAL, LFABP, isoprostane, IL-1, TNF-alpha, IL-6, and homocysteine) and a urinary sample (creatinine, albumin, urea, sodium, potassium, phosphate, proteins, urine test, urine cultures) will also be tested.

Additionally, the patient will undergo an exercise capacity assessment by means of the 6MWT, 5STS, and CPET tests and a psychosocial assessment using the following questionnaires: brief Illness Perception Questionnaire (BIPQ), Morisky Medication Adherence Scale 8 (MMAS-8), Chronic Illness Anticipated Stigma Scale (CIASS), Experience in Close Relationship Scale—Short Form (ECR-S), Health Care Relationship Trust Scale Revised (HCR-TS), Difficult Doctor–Patient Relationship Questionnaire 10 (DDPRQ-10), General Anxiety Disorder 7 (GAD 7), Patient Health Questionnaire 9 (PHQ-9), Edmonton Symptom Assessment System revised (ESAS-r), Severity of Dependence Scale (SDS), and Insomnia Severity Index (ISI). The qualities of life will be investigated using the 36-Item Short-form Survey (SF36). Finally, electrocardiogram (ECG), echocardiography, and cardiac MRI will be performed.

During the 12-month visit, the medical history will be updated, clinical parameters will be recorded, blood and urine tests will be repeated, the exercise capacity will be assessed using 6MWT, 5STS, and CPET, and the FASTEX Index will be also compiled.

At the 24-month visit, all the assessments and examinations from the baseline visit will be repeated. 

The statistical analysis will be performed using SPSS 21.0. The continuous variables will be presented as means and standard deviations, or medians and interquartile ranges depending on their distribution, which will be assessed using histograms and QQ plots. The nominal variables will be reported as frequencies and percentages. To determine any possible associations between SGLT2i and the variables of the primary and secondary outcomes, Pearson and Spearman tests will be used for continuous variables, the Fisher exact test for nominal variables, and the Student or Mann–Whitney tests for continuous and nominal variables. Finally, ANOVA tests will be performed for multicategory variables. A *p*-value of less than 0.05 will be considered statically significant for all tests. 

## 4. Conclusions

This study will be the first in the literature to evaluate the effect of Dapaglifozin in patients with albuminuria affected by CKD secondary to Fabry’s disease, primarily on renal outcomes (albuminuria and CKD progression) and, secondarily, on cardiovascular outcomes, exercise capacity, and quality of life. It will provide new compelling evidence on the use of SGLT2 inhibitors in the field of rare diseases.

## Data Availability

Not available. Further inquiries can be directed to the corresponding author.
